# Modification of the Lipid Profile of the Initial Oral Biofilm In Situ Using Linseed Oil as Mouthwash

**DOI:** 10.3390/nu13030989

**Published:** 2021-03-19

**Authors:** Anna Kensche, Marco Reich, Christian Hannig, Klaus Kümmerer, Matthias Hannig

**Affiliations:** 1 Clinic of Operative and Pediatric Dentistry, Medical Faculty Carl Gustav Carus, TU Dresden, Fetscherstr. 74, D-01307 Dresden, Germany; christian.hannig@uniklinikum-dresden.de; 2 Faculty of Sustainability, Institute of Sustainable and Environmental Chemistry, Leuphana University Lueneburg, Universitaetsallee. 1, C13, 21335 Lueneburg, Germany; marco.reich@leuphana.de (M.R.); klaus.kuemmerer@leuphana.de (K.K.); 3 Clinic of Operative Dentistry, Periodontology and Preventive Dentistry, University Hospital, Saarland University, Building 73, D-66421 Homburg, Germany; matthias.hannig@uks.eu

**Keywords:** pellicle, linseed oil, fatty acid, ultrastructure, in situ

## Abstract

Lipids are of interest for the targeted modification of oral bioadhesion processes. Therefore, the sustainable effects of linseed oil on the composition and ultrastructure of the in situ pellicle were investigated. Unlike saliva, linseed oil contains linolenic acid (18:3), which served as a marker for lipid accumulation. Individual splints with bovine enamel slabs were worn by five subjects. After 1 min of pellicle formation, rinses were performed with linseed oil for 10 min, and the slabs’ oral exposure was continued for up to 2 or 8 h. Gas chromatography coupled with electron impact ionization mass spectrometry (GC-EI/MS) was used to characterize the fatty acid composition of the pellicle samples. Transmission electron microscopy was performed to analyze the ultrastructure. Extensive accumulation of linolenic acid was recorded in the samples of all subjects 2 h after the rinse and considerable amounts persisted after 8 h. The ultrastructure of the 2 h pellicle was less electron-dense and contained lipid vesicles when compared with controls. After 8 h, no apparent ultrastructural effects were visible. Linolenic acid is an excellent marker for the investigation of fatty acid accumulation in the pellicle. New preventive strategies could benefit from the accumulation of lipid components in the pellicle.

## 1. Introduction

Bioadhesion and development of pathogenic biofilms on non-shedding solid surfaces are the key features for the initiation of caries, gingivitis, periodontitis as well as the increasing periimplantitis [1,2,3,4,5]. Due to this fact, one approach in developing new prophylactic mouthwashes is the search for agents that allow targeted and sustainable modulation of initial bioadhesion. The first step of bioadsorption in the oral cavity is the formation of the pellicle. This layer is composed of proteins, peptides, glycoproteins but also metabolites and lipids from the oral fluids [6,7,8]. Over the past decade, several in situ studies have demonstrated a noteworthy correlation between compositional and ultrastructural pellicle modifications and the variation of the pellicles’ protective properties at the tooth surface [9,10,11,12]. In this context, more and more food components and plant extracts have come into focus. Due to their biocompatibility, substances such as lipids and polyphenolic compounds might get integrated into or interfere with the natural adsorption processes at the tooth surface [12,13]. 

There are controversial data on the impact of oil mouthrinses on (bacterial) biofilm formation and erosion prevention under in vitro or in situ conditions [10,14,15,16,17,18]. Nevertheless, lipids are still of considerable interest due to their lipophilic character [7]. They account for 20% of the pellicle’s dry weight, but the lack of suitable and reliable methods to obtain the biological material in situ and to detect the specific components have in the past been clear limitations in this field of research [7,19,20]. Hydrophobic interactions are suggested to be a significant driving force for pellicle formation. The topical adsorption of lipophilic substances at the tooth surface after different lipid-containing mouthrinses was shown in a few in situ studies [7,10,17,19,21]. However, it remains uncertain whether and how the applied macromolecules are integrated into the in situ pellicle and whether the pellicle’s composition, structural pattern and properties can be altered sustainably, for example, by mouthrinses with edible oils. Precise detection of the applied lipid components is a prerequisite for corresponding investigations. 

Up to now, there are only a few studies on the lipid and fatty acid composition of the pellicle layer [20,21,22,23]. For that reason, we have already investigated the fatty acid content of the in situ pellicle with highly sensitive methods [19,24]. Based on gas chromatography coupled with electron impact ionization mass spectrometry, a pellicle-characteristic fatty acid profile with little interindividual variability was determined. Distinct differences were only detected regarding the total amount of adsorbed fatty acids, which also depended on the pellicles’ formation time [19]. Furthermore, the ultrastructure of the in situ pellicle and the initial biofilm had been characterized in detail by transmission electron microscopy [25,26,27]. Alterations were observed by TEM for a short period after mouthrinses with safflower oil [10]. In comparison to the native control, 2 h in situ pellicles that had been formed after the application of the oil mouthrinse appeared to be less electron-dense with a rather loose protein accumulation. Following this, an accumulation of lipid droplets at the in situ pellicle’s surface has only recently been visualized by environmental scanning electron microscopy for up to 9.5 h after rinsing with safflower oil [21]. 

Based on the knowledge from previous investigations, the present study aimed to clarify if rinses with a specific edible oil can modify the composition and ultrastructure of the pellicle layer sustainably. Linseed oil was selected for this purpose as it contains linolenic acid (18:3), which is not a physiological component of the saliva and accordingly served as a marker molecule. The obtained results will be relevant for the targeted development of new strategies in preventive dentistry that rely on the modulation of native bioadhesion processes. In this context, lipids could also serve as carriers that deliver protective substances to the tooth surface. In addition, valuable information may be gained about the dynamics of biomolecule adsorption at the tooth surface and the turnover of the in situ pellicle in general.

## 2. Materials and Methods

### 2.1. Subjects

As a preliminary investigation for this study, native saliva samples were collected from 12 subjects and the fatty acid profile was determined by GC-MS (see below). All volunteers had given their informed written consent about participation in the study. They underwent a clinical oral examination by an experienced dentist to ensure that they had no unrestored carious lesions, periodontal diseases, or an unphysiological salivary flow rate. Due to the high methodical effort, further in situ experiments regarding the effects of mouthrinses with linseed oil on saliva and the in situ pellicle were continued with five participants (aged 21–36). The study design was approved by the Ethics committee of the Medical Faculty, Technische Universität Dresden, Germany (vote: EK 475112016). Individual upper jaw splints were prepared for every participant to enable the exposure of bovine enamel specimens to the oral cavity for intraoral pellicle formation. Enamel slabs were obtained from the incisors of 2-year-old cattle, which are recognized as a suitable substitute for human enamel [28]. For in situ exposure, all enamel slabs were prepared as described in numerous previous publications [10,11,17,24]. The slabs’ surfaces were wet-ground and polished according to a standardized protocol with up to 4000 grit abrasive paper, and the resulting smear layer was then removed by ultrasonication in 3% NaOCl for 3 min. Two washing cycles in distilled water for 5 min were followed by disinfection of the specimens in 70% ethanol, all activated by ultrasonication. Finally, the bovine enamel specimens were stored in distilled water at 4 °C for up to 24 h before intraoral exposure.

### 2.2. Adopted Mouth Rinse and Rinsing Procedure

**Figure 1:**
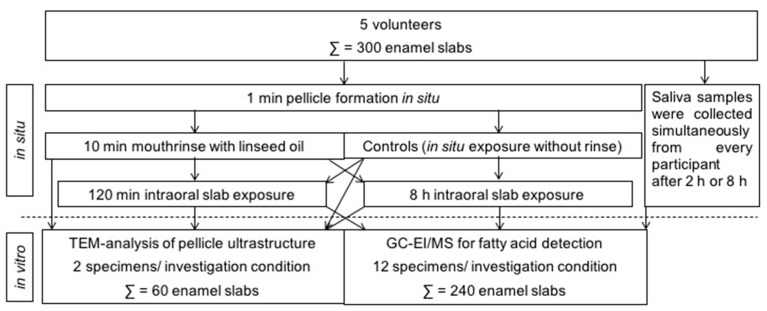
Overview of the experimental setup performed in this study. In situ pellicle and initial biofilm samples were obtained from 5 volunteers, who all underwent the different investigation conditions. In total, 300 enamel slabs were used in this study.

Different in situ experiments were carried out on separate days allowing a wash-out period of at least 48 h in which the subjects were asked to perform their regular oral hygiene. Two hours before an intraoral test period, the volunteers had to brush their teeth without toothpaste. In the following, no food or drinks other than water were to be consumed until the collection of in situ pellicle/biofilm samples. All participants had to carry their individual splints intraorally for either 120 min or 8 h overnight, with the enamel slabs attached in little cavities in the buccal and palatal region of the premolars and the first molar. Twelve enamel slabs per splint were exposed at a time to obtain sufficient in situ pellicle material necessary for the lipid analyses. TEM was performed on two pellicle samples per time of investigation and volunteer. The in situ rinsing protocol and exposure times were planned on the basis of earlier studies in this field [10,12,15,29], see Figure 1 for an overview of the experimental setup. After 1 min of pellicle formation in situ, mouthrinses were performed for 10 min with 10 mL of linseed oil. This specific edible oil was chosen as it contains linolenic acid (18:3), which under physiological conditions cannot be detected in the fatty acid profile of human saliva or salivary in situ pellicles. The fatty acid composition of the linseed oil used in this study is shown in Table 1. 

**Table 1:** Fatty acid composition of major fatty acids (in %, in relation to total fatty acids) in linseed oil with linolenic acid (18:3) being the predominant fatty acid. Comparison of the composition between oil used as a mouthrinse in this study (composition was determined by the GC-MS method described in this paper) and data provided in literature [30,31].

Fatty Acid	Linseed Oil (Used in this Study)	Linseed Oil (Literature Values)
16:0	4–9%	4–6%
18:0	2–9%	2–3%
18:1	12–14%	10–22%
18:2	12–14%	12–18%
18:3	58–66%	56–71%

Finally, all enamel slabs were removed from the splints and subjected to in vitro investigation methods. Simultaneously, unstimulated saliva samples were obtained from every participant. These samples were centrifuged at 6000× *g* for 10 min and sterile filtered (0.2 µm) before analysis. The same experimental runs were made without the use of oil. This allowed the collection of enamel slabs, as well as saliva, which had not been exposed to the oil and served as control.

### 2.3. GC-MS Analysis of the Fatty Acid Composition

The analysis of fatty acids in saliva and pellicle desorbates was performed as described in detail previously [24].

### 2.4. Sample Preparation

Tridecanoic and nonadecanoic acid were used as internal standards and were added to the desorbed pellicle samples before all sample preparation steps. The pellicle lipids were initially separated from the matrix by liquid/liquid extraction. For this step, an adapted Folch extraction procedure was used, in which the lipids were extracted from the desorbed pellicle sample with a mixture of methanol and chloroform. Rapid transesterification (1 h; 100 °C) of all fatty acids (FAs) containing lipids into fatty acid methyl esters (FAME) was carried out in methanol using concentrated HCl (35%, *w*/*w*) as an acidic catalyst. The FAMEs were then extracted by adding hexane and deionized water. The hexane phase was isolated, evaporated under a gentle stream of nitrogen, and the residue was redissolved in 0.1 mL of hexane. For GC-MS analyses, 1 µL of this solution was injected.

### 2.5. GC-MS (Instrumental Conditions)

GC/EI-MS was performed with an ISQ™ 7000 Single Quadrupole GC-MS system (Thermo Scientific, Dreieich, Germany). The injector was operated in splitless mode at 260 °C. For the separation of the target compounds, a TRACE™ TR FAME fused silica capillary column (30 m × 0.25 mm × 0.25 µm; Thermo Scientific, Dreieich, Germany) and helium as carrier gas was used with a constant flow of 1.5 mL/min. The oven temperature was programmed at 50 °C for 5 min, followed by an increase of 6.5 °C/min until the final temperature of 260 °C was reached and then held for 8 min. The electron energy was 70 eV, and the ion source temperature was set to 270 °C. A mass range of m/z 60–400 was recorded in full scan mode. Additionally, selected ion monitoring (SIM), recording fragment ions including m/z 74, m/z 79, m/z 81, and m/z 87 was performed throughout the run. 

In order to visualize the impact of mouthrinses with linseed oil on the ultrastructure of the in situ pellicle over time, transmission electron microscopy was performed on pellicle samples directly after the rinse, after 2 h or after 8 h of oral exposure, respectively. The findings were compared with control images of a characteristic native 3 min, 30 min, 120 min and an 8 h in situ pellicle. After completion of the oral exposure times, the enamel specimens were carefully rinsed with distilled water and fixed in 2.5% glutaraldehyde and 0.1 M cacodylate buffer for 1 h at 4 °C. Five washing cycles were performed in phosphate buffer before 1% osmium tetroxide was applied for 1 h to visualize the organic structures. The samples were then dehydrated in an ascending alcohol series and embedded in Araldite CY212 (Agar Scientific Ltd., Stansted, United Kingdom). All enamel residues were decalcified with 0.1 M HCl, and the remaining pellicle samples were re-embedded in Araldite. Ultrathin sections of the embedded pellicle samples were cut in series with a diamond knife in an ultramicrotome (Ultracut E, Reichert, Bensheim, Germany). The samples were mounted on pioloform coated copper grids (Plano, Wetzlar, Germany), and uranyl acetate and lead citrate were applied to enhance contrasts. TEM-analysis was performed at magnification from 20,000 to 100,000-fold using a TEM TECNAI 12 Biotwin (FEI, Eindhoven, The Netherlands). Representative images were taken during the investigation of the entire pellicle samples.

## 3. Results

### 3.1. Modification of the Pellicle’s Lipid Profile 

**Figure 2:**
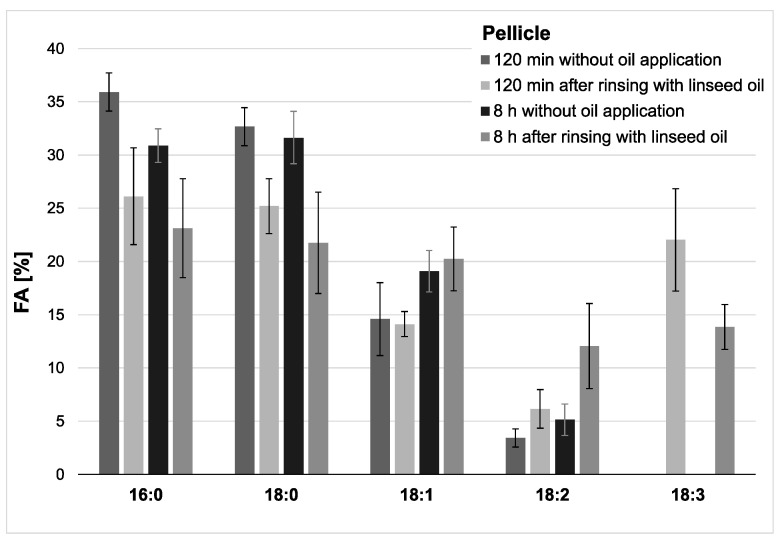
Percentage of the four major fatty acids and linolenic acid (18:3) in the pellicles. Measurements were performed after 2 h or 8 h of pellicle formation, either with or without linseed oil applied as a mouthrinse. Modifications of the in situ pellicle’s profile due to the oil application could be seen clearly at both investigation times. While linolenic acid could not be detected in the native controls at any time, it made approximately 22% of the total fatty acid composition after 2 h of pellicle formation and could still be detected in the 8 h biofilm samples (13%). It can be assumed that the fatty acids provided by the linseed oil mouthrinse were sustainably integrated into the in situ pellicle’s composition.

**Figure 3:**
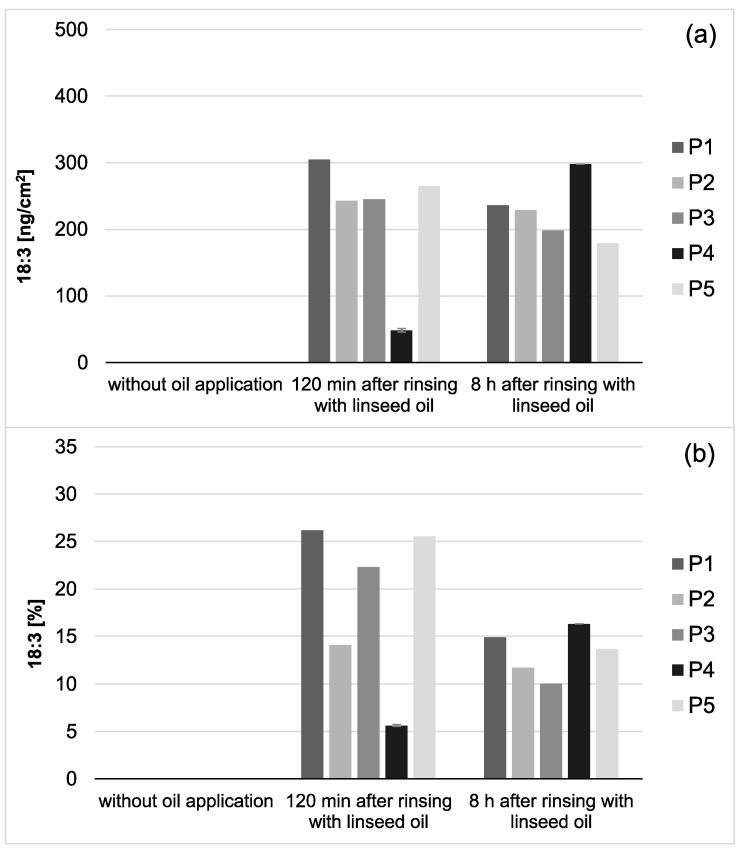
Total amount of 18:3 in the in situ pellicle (**a**) and proportion of 18:3 on the total fatty acid profile (**b**) of the 5 individual subjects (P1-5). In general, 18:3 was only detectable if mouthrinses with linseed oil had been applied. During the investigation period (2–8 h), the adsorbed amount of 18:3 appeared to be relatively consistent. However, after 8 h of biofilm formation, the proportion of 18:3 on the total fatty acid profile has decreased noticeably (**b**). This might be due to the salivary clearance, reduced availability of the specific fatty acid, and the resulting shift towards fatty acids naturally contained in saliva. Notably, smaller amounts of 18:3 were measured for participant 4 (P4) compared to the other subjects. However, the repetition of the measurements with new investigation material (*n* = 2) confirmed the previous results.

Modifications of the pellicle’s lipid profile were detected after gas chromatography coupled with electron impact ionization mass spectrometry (GC-EI/MS) was performed on pellicle samples that were collected in situ with or without having been exposed to mouthrinses with linseed oil. Linseed oil contains linolenic acid (18:3) (Table 1), which is not physiologically present in the human oral flora and thus serves as a reliable biomarker. Under consideration of already available data from previous investigations, the results focus on the detection of linolenic acid in comparison to the four major fatty acids palmitic (16:0), stearic (18:0), oleic (18:1) and linoleic acid (18:2), which generally account for approximately 90% of all investigated fatty acids in saliva and the in situ pellicle [19]. The analyzed pellicle samples after linseed oil rinsing showed a shift to a higher percentage of unsaturated fatty acids and a strongly increased proportion of linolenic acid (Figure 2 and Figure 3). Even 8 h after rinsing, the specific fatty acid 18:3 is still clearly detectable, which indicates that the lipids introduced through the mouthwash are sustainably integrated into the pellicle structure.

**Figure 4:**
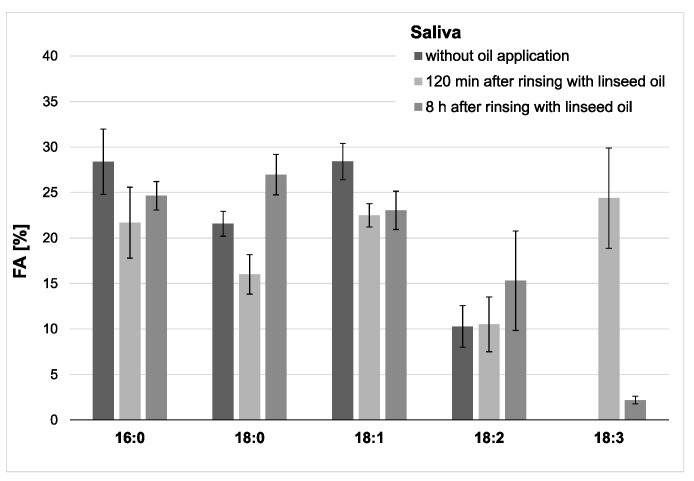
Percentage of the four major fatty acids and 18:3 in the saliva’s total fatty acid profile. The mean values and standard deviation are shown based on the data from five participants (*n* = 5). Saliva samples were collected 2 h and 8 h after the mouthrinse with linseed oil. Corresponding native saliva samples without oil application served as controls. The highest concentration of 18:3 was measured 2 h after the mouthrinse (approximately 24% of all fatty acids (FAs)). Even after 8 h, residues of 18:3 were still detectable. Linolenic acid was not found in any of the controls.

**Figure 5:**
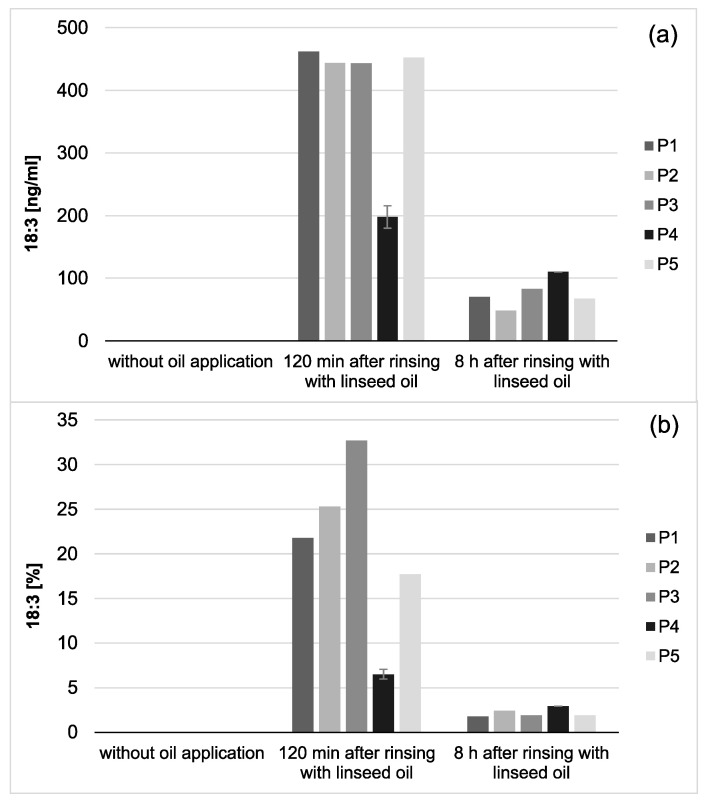
Total concentration of 18:3 in saliva (**a**) and proportion of 18:3 on the total salivary fatty acid profile (**b**) of five individual subjects (P1–5). 18:3 was only detectable if mouthrinses with linseed oil had been applied. Both, the total concentration as well as the proportion of 18:3 on the total fatty acid profile of the investigated saliva samples decreased considerably during the 8 h investigation period. Remarkably, small amounts of 18:3 could still be detected in the saliva samples 8 h after the linseed oil mouthrinse had been performed. Notably, smaller amounts of 18:3 were measured for participant 4 (P4) than for the other subjects. However, the repetition of the measurements with new investigation material (*n* = 2) confirmed the previous results.

In saliva, a high proportion of linolenic acid was found 2 h after the rinse, while the percentages of palmitic (16:0), stearic (18:0), oleic acid (18:1) were obviously reduced. The concentration and content of linolenic acid decreased notably within the examination period of 8 h after oil rinsing (Figure 4 and Figure 5). However, it is remarkable that under the given sampling conditions, even 8 h after oil rinsing, linolenic acid is still detectable in saliva. In the reference saliva samples (without preceding oil rinsing), linolenic acid was not detectable in any of the samples. 

In this study, no statistical analysis was performed. For the first time, the accumulation of an applied specific fatty acid (18:3) was transparently determined in the pellicle and in saliva with highly sensitive methods. The investigation focused on the detectability of linolenic acid after oil mouthrinses. Considering the one-to-one correspondence of present or absent linolenic acid in the investigated samples, statistical analyses of any kind were irrelevant.

### 3.2. Transmission Electron Microscopic Analysis

**Figure 6:**
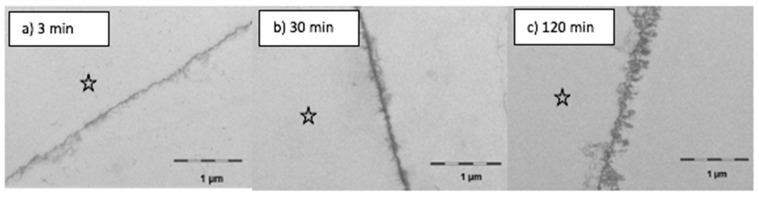
Transmission electron microscopy—control samples. The 3 min pellicle has a continuous electron dense basal layer with few granular structures (**a**). After longer oral exposure for 30 min (**b**) or 120 min (**c**) more granular and globular structures adsorb to the pellicle layer. The enamel was removed during the preparation of the samples; the former enamel side is marked with an asterisk.

Alterations of the pellicle’s ultrastructure were also temporarily seen by transmission electron microscopic analysis. The early stages of bioadsorption at the tooth surface generally create a characteristic image of a two-layer pellicle ultrastructure, as shown in Figure 6. Almost immediately, the enamel surface is covered by a relative uniform continuous 10–20 nm-thick electron-dense basal layer. As a result of prolonged oral exposure, more granular and globular structures adhere to the pellicle surface forming a second layer of variable thickness.

**Figure 7:**
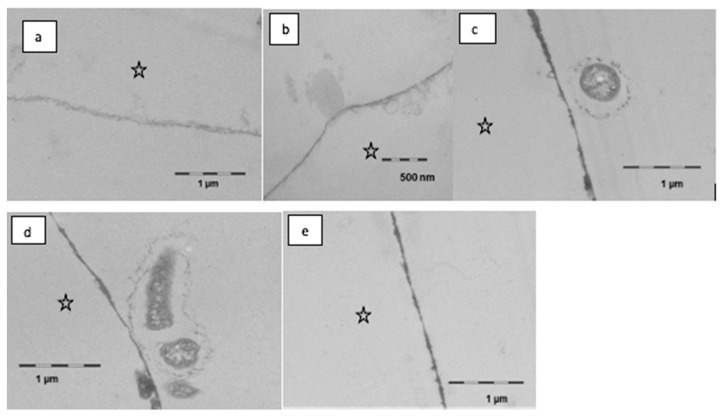
Transmission electron microscopy, in situ application of linseed oil for 10 min after 1 min of pellicle formation. Rinsing with the oil leads to a slight dissolution and thinning of the pellicle, partially the surface is smoother than in controls (**a**,**b**). Oil droplets were observed seldomly (**b**). In part, there was an optimized wetting of the surface in the sense of a subsurface pellicle. During further oral exposure for up to 120 min, a modified ultrastructure was still visible (**c**–**e**). The basal pellicle is thicker and more electron dense, sometimes pores in the enamel are filled with proteinaceous structures. Furthermore, first adherent bacteria were detectable; the cell wall of the bacteria is damaged (**c**,**d**). The enamel was removed during the preparation of the samples; the former enamel side is marked with an asterisk.

It was observed in this study that the formation and the appearance of this second more heterogeneous pellicle layer, to a certain extent, depended on the local accumulation of biological macromolecules at the tooth surface (Figure 7). Initially, rinsing with linseed oil for 10 min appeared to result in a slight dissolution or loosening of the pellicle’s ultrastructure, however creating a smooth surface. Oil droplets were randomly detected at the pellicle surface. On the other hand, in some parts, the protein adsorption to the enamel surface appeared to be optimized with proteinaceous structures covering even nano- and micro-pores like a subsurface pellicle. This slightly promoted affinity of proteins to the tooth surface and was still visible after 120 min of pellicle formation where the basal pellicle was thicker and more electron-dense than in the control. During these early stages of biofilm formation at the tooth surface, only a few bacteria were detected at the pellicle surface. Their morphology appeared to be affected by the linseed oil mouthrinse. The cell walls were damaged, and no cell division was observed.

**Figure 8:**
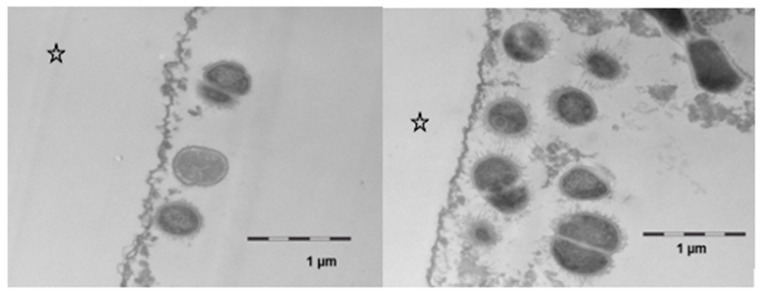
Transmission electron microscopy, in situ application of linseed oil for 10 min after 1 min of initial pellicle formation. The samples were kept in the oral cavity overnight for 8 h. There was a pronounced bacterial colonization, the adherent bacteria showed an intact ultrastructure and cell division was observed. No differences were observed as compared with controls. The enamel was removed during the preparation of the samples; the former enamel side is marked with an asterisk.

However, after 8 h of oral exposure, a considerable attachment of viable bacteria had occurred in several areas of the investigated samples (Figure 8). In comparison to the controls, more bacteria were found in the samples that had been exposed to the linseed oil mouthrinses. The cell walls appeared to be intact, and cell division as a sign of viability was detected. Even though the structure of this bacterial biofilm was still very loose, the bacteria’s interactions by bacterial fimbriae could be seen. Regarding protein accumulation or potential persistence of lipid components at this time, no notable ultrastructural differences were observed between the samples that had/ had not been exposed to the linseed oil mouthrinse. 

## 4. Discussion

In recent years, few in situ studies have been published that made a scientifically sound contribution to the incomplete knowledge about the involvement of lipids in initial bioadhesion processes at the tooth surface [10,15,19,21,32]. Clearly, as had already been suggested earlier, lipids and lipophilic molecules must be regarded as essential components of the in situ pellicle at various formation times [19,20,33]. At the same time, it appears that a modification of the lipid components provided in the oral cavity may have a detectable effect on the pellicle’s composition, structure, and functional properties [10,17,21]. However, only modern and highly sensitive analytical methods will give greater certainty about the actual integration of lipid components into the pellicle layer and their alteration due to remodeling processes. 

With this aim in mind, the performed application of gas chromatography coupled with electron impact ionization mass spectrometry (GC–EI/MS) as well as electron microscopic techniques (TEM) were suitable methods to detect and quantify (specific) lipid components in the in situ pellicle samples. At the time the study was conducted, micellar molecule aggregations had already been visualized in pellicle samples shortly after mouthrinses with edible oils and lipids containing food components [10,11]. Similar observations were made in the present investigation. In comparison to the native control, the ultrastructure of the in situ pellicle after mouthrinses with linseed oil showed a slightly more heterogeneous pattern during the first 120 min of pellicle formation (Figure 6, Figure 7 and Figure 8). It must be assumed that lipid components were adsorbed to the pellicle layer and that the resulting molecular interactions had a notable effect on the adsorption and distribution of proteins at the tooth surface. Most biological lipids (phospholipids, sphingolipids, glycolipids), as well as free fatty acids, are amphiphilic molecules with hydrophilic and lipophilic properties [20]. On the one hand, hydrophobic repellent forces of the long hydrocarbon chains of fatty acids could be the reason for the slightly loosened ultrastructure of the second pellicle layer. On the other hand, in aqueous solution, fatty acids tend to form micellar aggregations (Figure 7b). It is even conceivable that these acted as carrier systems that delivered non-polar proteins to the tooth surface [22]. Two hours after the mouthrinse with linseed oil, a subsurface pellicle was occasionally detected, and parts of the basal pellicle appeared slightly more electron dense. The affinity of lipids to bind amphiphilic molecules or proteins, respectively, and to therefore alter the ultrastructure of the in situ pellicle had been described earlier in case of mouthrinses with bovine milk [17]. However, considering the dynamics of dental biofilm formation and the salivary clearance, it remained uncertain if these pellicle modifications had any sustainable effect for more than 2 h. Furthermore, details on the type of lipids or fatty acids accumulated in the pellicle were preferable to possibly understand their impact on pellicle and initial bacterial biofilm formation. 

Characteristic distributions of major fatty acids were detected in both physiological saliva and pellicle samples by GC–EI/MS. A comparatively higher proportion of saturated fatty acids in the pellicle demonstrates the selective adsorption of salivary components on the tooth surface. During maturation of the in situ pellicle overnight, the fatty acid profile appeared to be relatively consistent. 

Choosing linseed oil for the mouthrinse allowed the definite detection of oil-associated lipid accumulations in all investigated saliva and pellicle samples by GC–EI/MS. Linolenic acid (18:3) is not included in the physiological fatty acid profile of the oral fluids, and its detection in the pellicle could reliably be correlated with its absorption from the oil mouthrinse. Therefore, this specific fatty acid served as a suitable biological marker for the tracking of bioadhesion processes and their durability at the tooth surface. Based hereon, it was shown that the mouthrinse initially induced a shift of the major fatty acid composition to a higher percentage of unsaturated fatty acids and a remarkable accumulation of linolenic acid in both the saliva and pellicle samples (2 h). As already indicated above, the higher percentage of unsaturated fatty acids and the high reactivity of their double bonds might have influenced the surface wettability and the molecular interactions during pellicle formation. As expected, after 8 h, the mouthrinse alterations of saliva’s major fatty acid profile were almost eliminated due to the salivary clearance. The content of linolenic acid decreased by approximately 85% from 2 to 8 h after the rinse, yet linolenic acid was still traceable (Figure 3). Interestingly, pellicle maturation during these hours only depended to a certain extent on the availability of lipid components provided by the saliva. Oil-induced modifications of the pellicle’s fatty acid profile appeared to be notably more, continuing with a considerably lower turnover after 8 h of initial biofilm formation (~35% less linolenic acid from 2 to 8 h after the oil-rinse, Figure 2). Simultaneously, augmentation of pellicles’ thickness and bacterial adhesion were shown in the electron microscopic analyses. However, the ultrastructural pattern did not differ notably from the native controls.

Concerning a potential modification of functional pellicle properties in terms of erosion prevention or bacterial biofilm formation, so far, there is no undoubted evidence that mouthrinses with edible oils have a convincing health-promoting effect [10,14,15,16,20]. Nevertheless, the present TEM-images still indicate that components of the linseed oil initially affected the morphology of oral bacteria (Figure 7). Damage to the cell wall integrity of bacteria can be a sign of reduced viability. Taking in vitro studies into account, it can be assumed that certain free fatty acids, possibly also linolenic acid, had an antibacterial effect on salivary bacteria [34,35]. Fatty acids might have affected the integrity of bacteria’s cell membranes and interfered in metabolic processes which could explain the deformed morphology of some bacteria shortly after the mouthrinse. However, these effects were only seen in the first 2 h after the mouthrinse. It is also conceivable that bacteria’s adhesion on the pellicle was influenced by the distribution pattern of lipid components in the in situ pellicle. Looking at the TEM-images after 8 h of biofilm formation, bacterial adhesion appeared to have proceeded unhampered, if not had even been enhanced by lipid components, respectively. No clear difference could be seen between the controls and the samples that had been exposed to the linseed oil mouthrinse regarding the morphology of the adhering bacteria. This is in line with previous in situ investigations that did not measure a significant effect of edible oil mouthrinses on the adherence or live/dead ratio of bacteria at the tooth surface after 8 h of biofilm formation [15]. Further investigations in this field will be necessary. 

## 5. Conclusions

Using highly sensitive analytical methods, this study clarified for the first time that mouthrinses with edible oil can alter the fatty acid profile of the in situ pellicle and the initial bacterial biofilm sustainably. In this context, linolenic acid was identified as a valuable biological marker as it is not present in the fatty acid profile of native saliva or in situ pellicles. The results obtained from this investigation provide important information about the kinetics of pellicle formation at the tooth surface in general. However, further research should focus on the relevance of lipid components’ molecular characteristics and molecular interactions for the accumulation of proteins and bacteria at the tooth surface, respectively. The modification of lipid components in the in situ pellicle remains an interesting approach to alter and possibly improve the physiological functional properties of the in situ pellicle.

## Data Availability

All relevant data are contained in the article.

## Abbreviations

GC-EI/MS	Coupled with electron impact ionization mass spectrometry
TEM	Transmission electron microscopy
18:3	Linolenic acid
16:0	Palmitic acid
18:0	Stearic acid
18:1	Oleic acid
18:2	Linoleic acid
NaOCl	sodium hypochlorite
NaOCl	sodium hypochlorite
FAME	Fatty acid methyl esters
HCl	Hydrochloric acid
